# Competition of Moiré Network Sites to Form Electronic Quantum Dots in Reconstructed MoX_2_/WX_2_ Heterostructures

**DOI:** 10.1021/acs.nanolett.3c04427

**Published:** 2024-01-31

**Authors:** Isaac Soltero, Mikhail A. Kaliteevski, James G. McHugh, Vladimir Enaldiev, Vladimir I. Fal’ko

**Affiliations:** † Department of Physics and Astronomy, University of Manchester, Oxford Road, Manchester M13 9PL, United Kingdom; ‡ National Graphene Institute, University of Manchester, Booth Street East, Manchester M13 9PL, United Kingdom; § Henry Royce Institute for Advanced Materials, University of Manchester, Oxford Road, Manchester M13 9PL, United Kingdom

**Keywords:** 2D materials, twistronics, heterobilayers, lattice relaxation, quantum dots

## Abstract

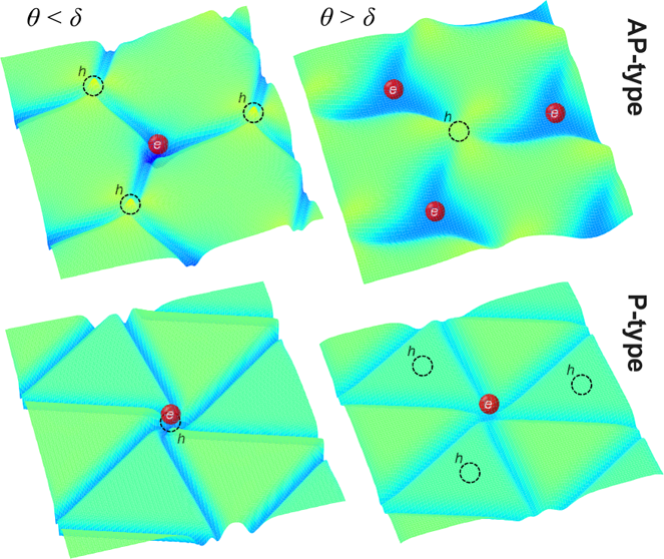

Twisted bilayers of two-dimensional semiconductors offer a versatile platform for engineering quantum states for charge carriers using moiré superlattice effects. Among the systems of recent interest are twistronic MoX_2_/WX_2_ heterostructures (X = Se or S), which undergo reconstruction into preferential stacking domains and highly strained domain wall networks, determining the electron/hole localization across moiré superlattices. Here, we present a catalogue of options for the formation of self-organized quantum dots and wires in lattice-reconstructed marginally twisted MoX_2_/WX_2_ bilayers with a relative lattice mismatch δ ≪ 1 for twist angles ranging from perfect alignment to θ ∼ 1°. On the basis of multiscale modeling taking into account twirling of domain wall networks, we analyze bilayers with both parallel and antiparallel orientations of their unit cells and describe crossovers between different positioning of band edges for electrons and holes across moiré superlattices when θ < δ and θ > δ.

Among various heterostructures of two-dimensional (2D) materials, bilayers of same-chalcogen transition metal dichalcogenides (TMDs) MoX_2_/WX_2_ (X = Se or S) stand out because of a uniquely small mismatch of monolayer lattice constants (∼0.3% for MoSe_2_/WSe_2_ and ∼0.2% for MoS_2_/WS_2_). An almost lattice matching at the interface promotes reconstruction of the moiré superlattice (mSL) in a bilayer into domains where monolayer lattices conform to each other, separated by domain wall networks (DWNs) that absorb hydrostatic and shear (for small angle twisted bilayers) strain. Such reconstruction was observed in bilayers assembled by 2D crystal transfer^1−6^ and synthesized using chemical vapor deposition (CVD).^7^ For bilayers with a larger twist, lattice reconstruction is less prominent; however, those bilayers also feature moiré superstructures, which have been extensively investigated in the context of localization of charge carriers and interlayer excitons in specific stacking regions of the mSL.^8−14^

In terms of the electronic properties of MoX_2_/WX_2_, which are type II heterostructures,^15^ the mapping^4,16,17^ of conduction/valence band edge variation across the mSL appears to be sensitive to the strain developed upon a bilayer’s lattice reconstruction. In particular, for marginally twisted bilayers, the formation of DWNs absorbs the dilation/compression and torsion required to adjust the two crystalline lattices within domains (in terms of the lattice mismatch of MoX_2_ and WX_2_ and an interlayer twist).^1−7^ Typical structures are illustrated in the top panels of Figure 1 by the maps of hydrostatic strain in MoSe_2_: honeycomb for bilayers with the antiparallel (AP) orientation of unit cells and triangular for the parallel (P) orientation. For AP bilayers, hexagonal domains correspond to 2H stacking, while corners of the DWN feature energetically unfavorable XX (chalcogen over chalcogen) and MoW (metal over metal) stacking domains.^17−19^ P bilayers feature MoX (metal over chalcogen) and XW (metal under chalcogen) stacking domains, with XX stacking DWN nodes.^17−19^

**Figure 1:**
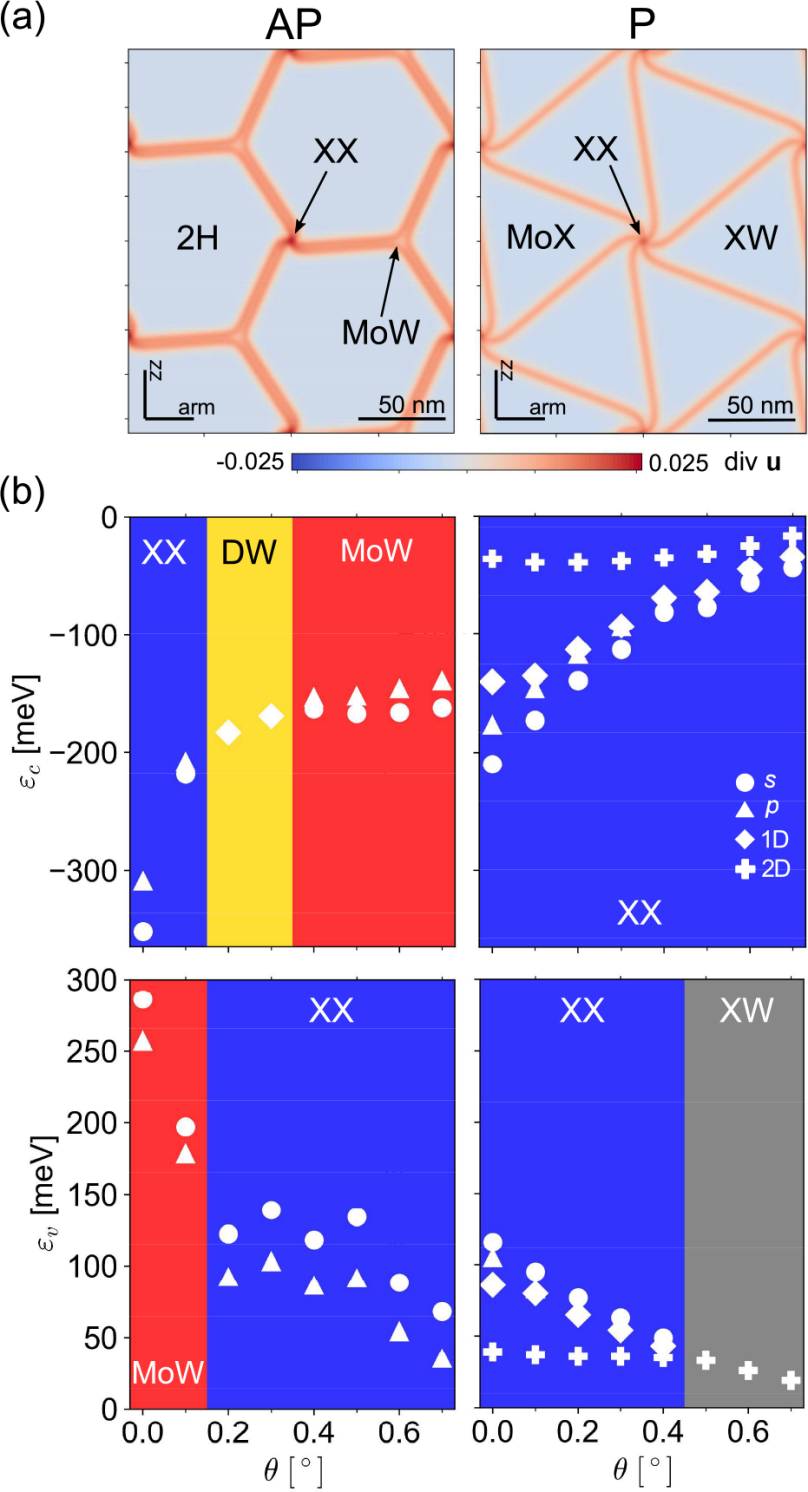
Crossover between different confinement regimes for electrons and holes on a moiré superlattice in marginally twisted AP-MoSe_2_/WSe_2_ (left) and P-MoSe_2_/WSe_2_ (right) bilayers. (a) Hydrostatic strain maps in the Mo layer for θ = 0°. Stacking configurations for domains and the zigzag (zz) and armchair (arm) crystallographic axes of the monolayers are indicated in each map. (b) Binding energies for electrons (ε_c_) and holes (ε_v_) trapped in the parts of DWN identified for each twist angle interval and marked by blue/red for XX/MoW nodes, yellow for one-dimensional (1D) domain walls, and gray for 2D XW domain areas (this color coding will be used in other figures). Circles and triangles are used for s and p type quantum dot-bound states, respectively. Diamonds mark binding energies of 1D domain wall states; crosses show the difference between band edges in MoX and XW domain areas.

In this paper, we demonstrate a high sensitivity of the band edge properties of marginally twisted (small angle |θ| < 1°) MoX_2_/WX_2_ bilayers to the lattice reconstruction and DWN formation. Recently, it has been noted that, while the nodes of such DWNs represent the areas of the largest strain, both hydrostatic (lattice dilation in MoX_2_ vs compression in WX_2_) and shear,^5,19−21^ the higher energetic cost of hydrostatic strain can be reduced by transforming some dilation/compression into shear achieved via “twirling” of DWN,^22−25^ as marked on the maps in Figure 1. Here, we highlight the role of hydrostatic strain, because it causes stronger band edge shifts than shear deformations^21,26−28^ (inset of Figure 2), hence affecting electron and hole confinement across the reconstructed moiré structure. The highest values for hydrostatic strain (corresponding to XX nodes), quantified in Figure 2 as the strain tensor trace (*u*_*ii*_ ≡ div **u**), reveal its predominant role for nearly aligned heterobilayers.

**Figure 2:**
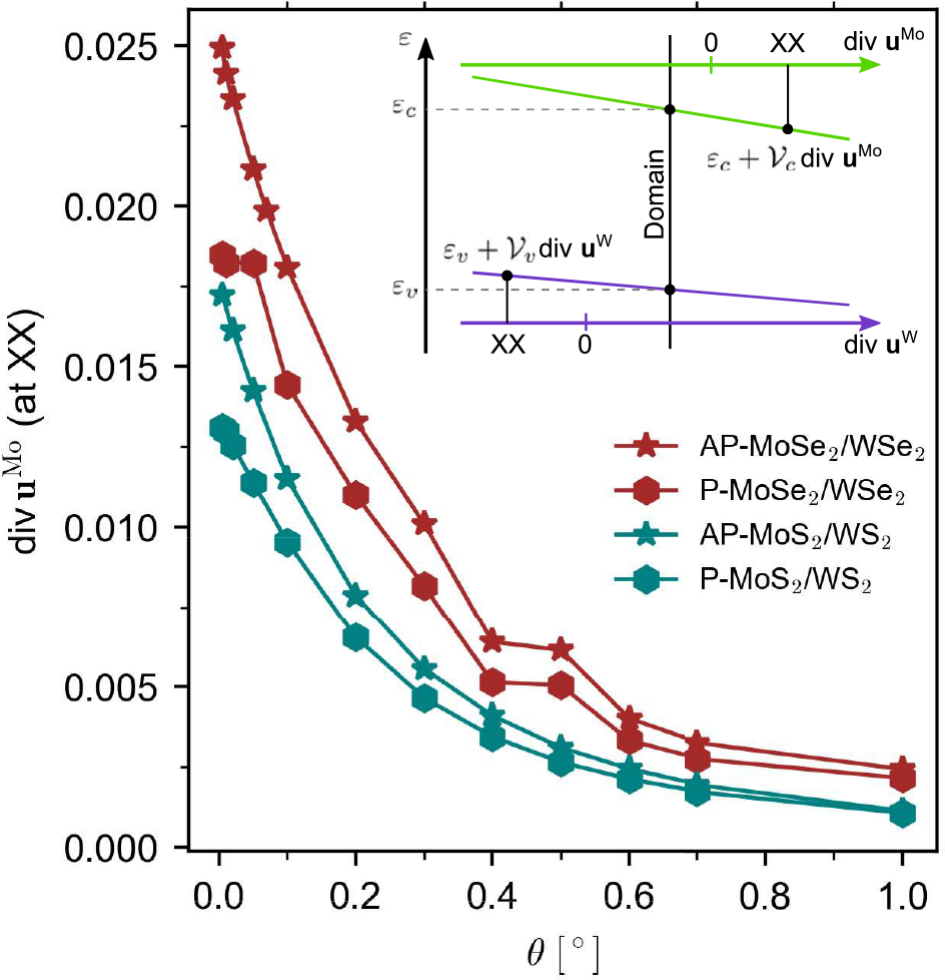
Twist angle dependence of hydrostatic strain in the Mo layer, div **u**^Mo^, at the XX nodes of AP-MoX_2_/WX_2_ (stars) and P-MoX_2_/WX_2_ (hexagons). Values in heterobilayers with different chalcogens (X = Se of S) are shown with brown or turquoise symbols, respectively. The inset shows the linear shift of K-valley conduction (green) and valence (purple) band edges in the MoX_2_ and WX_2_ layers, respectively. The solid black line indicates the value of hydrostatic strain in individual layers inside stacking domains.

The resulting variety is illustrated for MoSe_2_/WSe_2_ in the bottom panels in Figure 1. In particular, for an almost perfectly aligned AP bilayer (θ < 0.15°), corresponding to θ[rad] < δ, a relative lattice mismatch between MoX_2_ and WX_2_, we find that electrons and holes are localized at quantum dots at the opposite corners of hexagonal DWNs, with binding energies (denoted as ε_c_ and ε_v_ for electrons and holes, respectively) of both s- and p-like orbitals in the range of 200–300 meV, as shown in Figure 1. For slightly larger twist angles, θ > 0.15°, we find that the area of confinement for valence band holes relocates from the MoW to XX corners of the DWN, whereas for the conduction band electrons, the quantum dot at the XX node blends into the surrounding stretches of domain walls for an intermediate range of angles (0.15° < θ < 0.35°), which, now, can be considered as quantum wires. With a further increase in the twist angle (θ > 0.35°), electrons are eventually located at the MoW cornes of the DWN.

This contrasts with the very different systematics emerging for P-MoSe_2_/WSe_2_, where, when θ < 0.45°, the lowest-energy states for both electrons and holes appear to be at the XX nodes of the triangular DWN network. The right-hand side panels in Figure 1 indicate that, while for the larger angles the “XX quantum dots” persist for the conduction band electrons (bound states only 10–20 meV below the lowest 1D band inside the domain wall “wires”), the valence band holes become deconfined into the XW stacking domain areas, where the band edge is promoted by interlayer charge transfer,^17,29−33^ as compared to MoX domains. As discussed in detail in the rest of the paper, similar scenarios also appear in MoS_2_/WS_2_.

Also, we note that hydrostatic strain differentiates between heterobilayers and the previously studied homobilayers^5,19,20^ where strain near the DWN is predominantly shear, and conduction and valence band edges are modulated by potentials due to piezocharges. In heterobilayers with θ[rad] < δ, the domain walls can be classified as interlayer edge dislocations, in contrast to twisted homobilayers where domain walls are essentially interlayer screw dislocations. However, for twist angles θ[rad] > δ, the hydrostatic strain component in DWN is reduced (Figure 2), making domain walls more similar to screw dislocations and resulting in the same positioning of quantum dots for electrons and holes as in homobilayers.

## Methods

The multiscale modeling approach implemented in this study (see the Supporting Information) consists of the following steps.

(i) To describe the variation of the local stacking across the moiré pattern, we use a model developed in refs (17) and (19), which combines the microscopically computed [using density functional theory (DFT)] stacking-dependent adhesion energies between the MoX_2_ and WX_2_ crystals with an elasticity theory description of intralayer deformations.
(ii) The computed deformation fields in each layer are used to describe the energies of the electron (in MoX_2_) and hole (in WX_2_) band edges at the K points of the TMD Brillouin zone. It should be mentioned that both hydrostatic and shear deformations have opposite signs in MoX_2_ and WX_2_, and twirled DWNs feature less hydrostatic strain than do geometrically straight network structures.^18,21^
(iii) In the analysis of band energies, we account for both the band edge shift due to hydrostatic strain^21,26−28^ and piezocharges due to shear strain, both computed locally across the domain network and plotted as 2D maps over the moiré supercell to identify the potential confinement areas for charge carriers near the DWN nodes.
(iv) Having identified those confinement areas, we use an effective mass approximation for electrons and holes to compute their respective quantum dot spectra.

The lattice structure of the reconstructed bilayer is described employing 2D displacement fields **u**^(Mo/W)^(**r**), which determine the local lateral offset between the two crystals
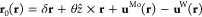
1where δ ≡ 1 – *a*_W_/*a*_Mo_ is a relative lattice mismatch between MoX_2_ and WX_2_, defined in terms of their respective lattice parameters *a*_Mo_ and *a*_W_. Note that for each local stacking configuration we use the optimal interlayer distance, *d*(**r**_0_)^19^ (which corresponds to the minimal of adhesion energy for each **r**_0_). Then, we minimize the total energy of the bilayer over the moire supercell, taking into account both elastic and adhesion energy contributions (see the Supporting Information for details). Two representative solutions for strains in P and AP bilayers are displayed in the top panels of Figure 1 as color maps of the developed hydrostatic strain in the MoX_2_ layer, div **u**^Mo^. A small hydrostatic strain inside domains compensates for the small lattice mismatch between the layers (div **u**^Mo^ ≈ −δ, and div **u**^W^ ≈ δ); the corresponding strain energy in both layers is fully compensated by the increase in adhesion energy,^19^ as proven by the observation of thermally annealing MoSe_2_/WSe_2_ bilayers into fully commensurate heterostructures.^34^ We emphasize that only hBN-encapsulated TMD heterobilayers are considered in this analysis, which allows us to neglect the formation of bulges and out-of-plane defects.

To include hydrostatic strain in the conduction and valence band edge shift analysis, we performed DFT calculations using the Quantum ESPRESSO code^35^ (see the Supporting Information). In all cases, band edge shifts can be approximated by  with the following DFT-computed values:  eV,  eV,  eV, and  eV. These values are in agreement with several previous *ab initio* studies.^21,26−28^ This hydrostatic strain effect is incorporated into the band energy profiles (see the Supporting Information for details)
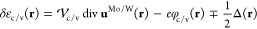
2where φ_c/v_(**r**) is a strain-induced piezoelectric potential (calculated for an hBN-encapsulated bilayer) and Δ(**r**) is a stacking-dependent energy shift due to a weak interlayer polarization (largely varying across the reconstructed moiré supercell in P and weakly in AP bilayers^17^). This determines the effective quantum dot profiles for the conduction and valence bands, implemented as databases for heterostructures with different twist angles, all of which have a *C*_3_ symmetry for both P and AP alignment. Then, we use brute force diagonalization to analyze the confinement of electrons and holes (see the Supporting Information for details).

## Band Edges in AP-MoX_2_/WX_2_

**Figure 3:**
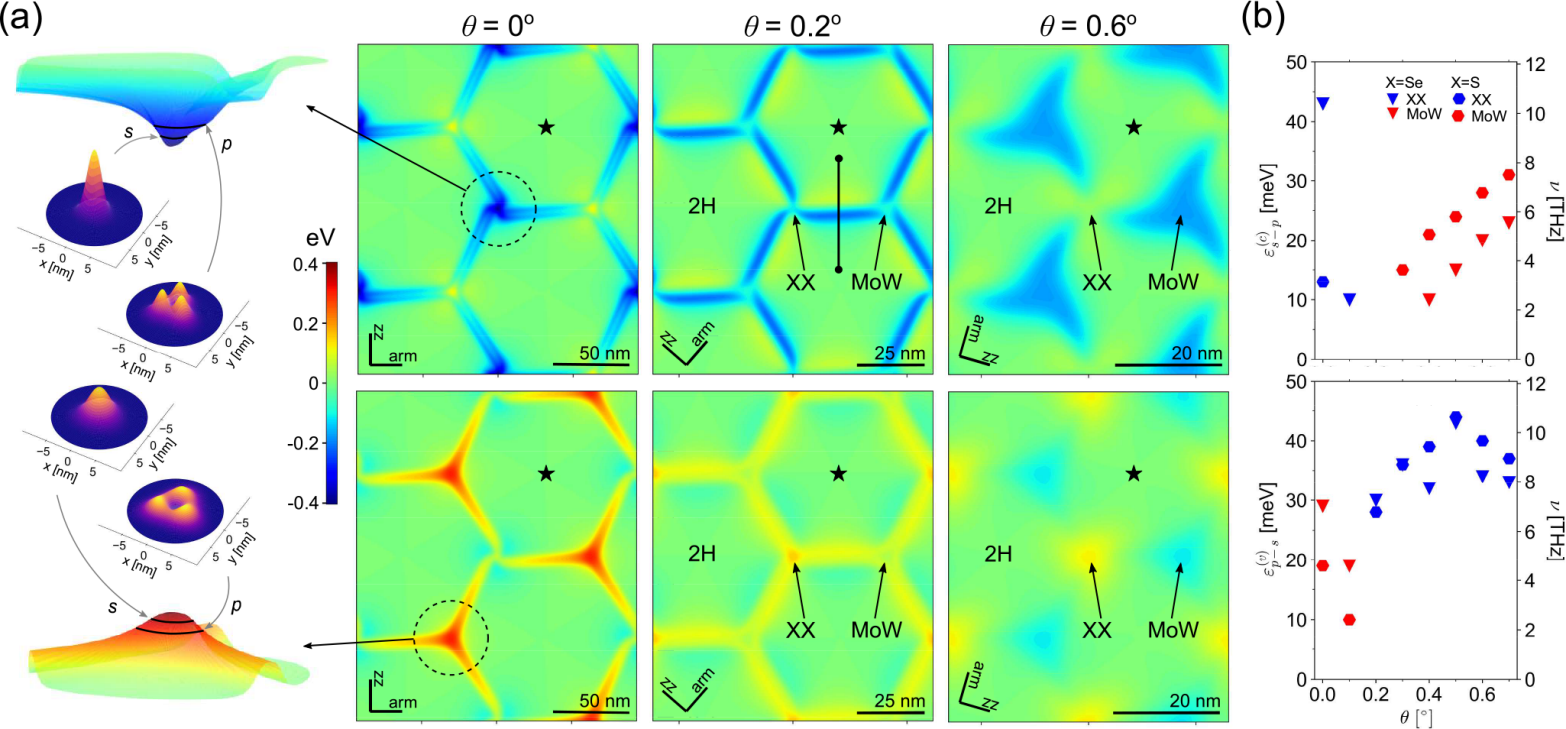
Conduction and valence band edge profiles in marginally twisted AP-MoSe_2_/WSe_2_ illustrating the crossover between different confinement regimes (as summarized in Figure 1). (a) 2D maps show the modulation of conduction (top) and valence (bottom) band edges for different twist angles. All energies are calculated from the corresponding c/v band edge energy in the middle of 2H stacking domains (marked by stars), and each map is rescaled according to its corresponding moiré periodicity (scale bar and crystallographic axes of the monolayers are indicated in all maps). The segment in the intermediate conduction band edge map indicates the one-dimensional potential profile for the calculation of bound states in domain walls. The left panel shows the conduction and valence quantum dot profiles for θ = 0°, with squared moduli of s and p states indicated in each case. (b) Twist angle dependence of the energy of intralayer s–p transitions, ε_s–p_ (and their corresponding frequency, ν), for quantum dots in the XX (blue symbols) and MoW nodes (red symbols) in the conduction (top) and valence (bottom) band edges of AP-MoX_2_/WX_2_ structures. Triangles and hexagons are used for X = Se and S compounds, respectively. The change of symbol colors reflects the swapping of band edge positions for electrons and holes around twist angle θ ∼ δ.

In this section, we discuss bound states in AP-MoX_2_/WX_2_ (X = Se or S), where the inner areas of hexagonal shape domains are occupied by a 2H type stacking (top layer metals over bottom layer chalcogens, and vice versa). The conduction and valence band edge profiles, calculated with respect to the center of the 2H stacking domains, are shown in Figure 3. Below, we discuss separately electron and hole confinement.

### Quantum Dots and Wires at Conduction Band Edges (electrons)

For closely aligned AP-MoSe_2_/WSe_2_ structures (θ < 0.15°), the joint effect of hydrostatic strain and piezoelectric potential results in deep quantum wells at the XX nodes, forming quantum dot states in those regions, with the energies of bound s and p states plotted in Figure 1 with circles and triangles, respectively. In an intermediate range of angles, 0.15° < θ < 0.35°, the reduction of hydrostatic strain and the inversion of the sign of piezoelectric charge (see the Supporting Information) result in the disappearance of the local energy minima in the DWN nodes (see Figure 3), pushing conduction band edges into domain walls, where 1D bound states can be viewed as stretches of quantum wires. Lowest subband energies for such quantum wires are shown in Figure 1 with diamonds. For larger twist angles, θ > 0.35°, piezopotential plays the predominant role, producing quantum dots in the MoW corners, with the energies of bound states represented as circles and triangles in Figure 1.

We note that approaching the interval 0.15° < θ < 0.35° with the band edge-forming domain wall quantum wires, s and p quantum dot-bound states in XX (θ ≈ 0.1°) and MoW (θ ≈ 0.35°) become nearly degenerate. However, a further increase in the twist angle promotes MoW quantum dots, leading to a lift of the degeneracy and the reappearance of s and p bound states.

### Quantum Dots in MoW and XX Corners at the Valence Band Edge (holes)

For the valence band in a bilayer with θ[rad] < δ, the piezopotential contribution to the band edge energy negates the confining effect of hydrostatic strain at the XX nodes. Instead, it produces distinct quantum dots in the MoW areas (see Figure 3) with binding energies of s and p states shown in Figure 1 with circles and triangles, respectively. The reduction of hydrostatic strain for θ[rad] > δ leaves the piezopotential as the dominant effect. Therefore, the change in the sign of piezocharges at θ[rad] ≈ δ shifts the quantum dot position toward the XX nodes, where the binding energies (of holes) are smaller.

The scenario of electron and hole confinement by DWNs in marginally twisted AP-MoS_2_/WS_2_ is similar (see the Supporting Information). The only qualitative difference is that the electron confinement at XX nodes is fragile against electrons leaking into domain wall (DW) quantum wires.

The overall evolution of the band edge landscape across the moiré supercell is shown in Figure 3 for both conduction and valence band electrons, with typical structural orbitals of s and p states attributed to the quantum dots. While the binding energies of such quantum dots states are plotted in Figure 1 (see the Supporting Information for binding energies in AP-MoS_2_/WS_2_), two right-hand side panels in Figure 3 quantify the excitation spectra of intradot intraband s–p transitions for the confined electrons that are optically active in the terahertz range.

## Band Edges in P-MoX_2_/WX_2_

**Figure 4:**
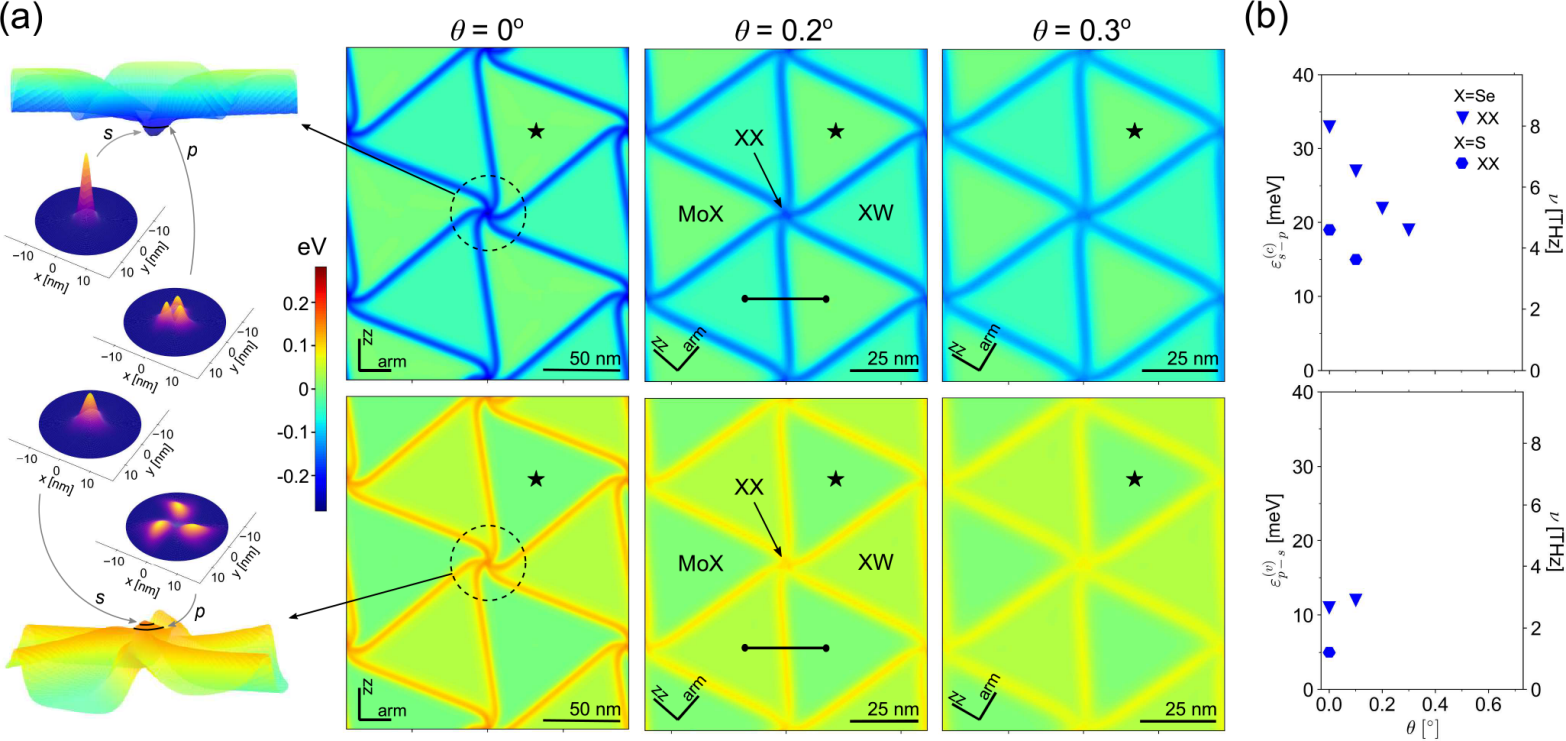
Crossover between different confinement regimes for conduction and valence band edges in marginally twisted P-MoSe_2_/WSe_2_ (as summarized in Figure 1). (a) 2D maps show the modulation of the conduction (top) and valence (bottom) band edges for different twist angles. All energies are calculated from the corresponding c/v band edge energy in the middle of MoX stacking domains (marked by stars), and each map is rescaled according to its corresponding moiré periodicity (the scale bar and crystallographic axes of the monolayers are indicated in all maps). The segments in the intermediate maps indicate the one-dimensional potential profiles for the calculation of bound states in domain walls. The left panel shows the conduction and valence quantum dot profiles for θ = 0°, with squared moduli of s and p states indicated in each case. (b) Twist angle dependence of the energy of intralayer s–p transitions, ε_s–p_ (and their corresponing frequency, ν), for quantum dots in the XX nodes in the conduction (top) and valence (bottom) band edges of P-MoX_2_/WX_2_ structures. Triangles and hexagons are used for X = Se and S compounds, respectively. The absence of transitions for larger angles is a consequence of blending of the p quantum dot states into domain walls.

In this section, we discuss bound states in P-MoX_2_/WX_2_ (X = Se or S), where DWNs separate triangular domains with MoX (top layer metals over bottom layer chalcogens) and XW (top layer chalcogens over bottom layer metals) stacking. The obtained band edge profiles are shown in Figure 4, where energies for the conduction (valence) band were calculated with respect to the highest (lowest)-energy stacking domain, which corresponds to the MoX configuration. For highly aligned P-MoSe_2_/WSe_2_, both conduction and valence band edge maps have their minimum value at the DWN intersections (XX nodes), with constant depth channels along domain walls, generated mainly by hydrostatic strain. Moreover, the energy difference between stacking domains, introduced by interlayer charge transfer, produces triangular quantum boxes in the XW stacking domains. As the twist angle is increased, there is a progressive reduction of the energy minima and the depth of the channels compared to the energy in stacking domains. This is driven by the drop in div **u**, which is the only contribution along the DWN [piezopotential and charge transfer contributions are distributed over the stacking domains (see the Supporting Information)]. Due to the difference in the magnitude of hydrostatic strain effects on the conduction and valence bands, below, we discuss electron and hole confinement separately.

### Quantum Dots and Wires at Conduction Band Edges (electrons)

In small angle P-MoSe_2_/WSe_2_ (θ < 0.35°), the strong effect of hydrostatic strain leads to the coexistence of quantum dots, wires, and 2D boxes, whose bound state energies are shown in Figure 1. Quantum dots at XX nodes hold bound states for the full range of twist angles studied, although p states persist up to only θ = 0.3°, spreading along the DWN for larger twist angles.

### Quantum Dots and Wires at Valence Band Edges (holes)

Despite having a structure qualitatively identical to conduction band profiles, valence band edges in P-MoSe_2_/WSe_2_ acquire shallower confinement regions with quantum dot- and wire-bound states up to θ ≈ 0.45° (p states obtained for only θ < 0.15°), with energies shown in Figure 1. Increasing the twist angle (θ > 0.45°) leads to relocalization of the lowest-energy state for holes to the XW stacking domains. This crossover from quantum dots and wires to 2D confined states is described in Figure 1.

The results for P-MoS_2_/WS_2_ exhibit a slightly modified picture. In this case, electrons escape the DWN for θ > 0.65° (see the Supporting Information), producing a weakly confined 2D state. Furthermore, holes reveal bound state energies for only a small range of angles (θ < 0.25°), hosting p states only for the aligned structures (θ = 0°). For both MoSe_2_/WSe_2_ and MoS_2_/WS_2_ bilayers, the quantum dots in XX corners support s and p bound states for only almost aligned crystals, and in the two right-hand side panels in Figure 4, we quantify the energies of optically active intradot intraband s–p transitions.

## Discussion and Conclusions

Overall, the reported analysis of the band edge landscapes in lattice-reconstructed marginally twisted bilayers of same-chalcogen TMDs suggests an entertaining scenario that unfolds over a small range of misalignment angles, |θ| ≤ 0.7°, for the localization of electrons and holes across the moiré supercell.

In particular, in perfectly aligned AP bilayers, we find distinct quantum dots in the opposite XX and MoW corners for electrons and holes, respectively, which swap with an increase in the twist angle beyond θ[rad] > δ. This means that a very small variation in the heterostructure assembly conditions may qualitatively alter the nature of self-organized quantum dots in the reconstructed moiré pattern. Theoretically, the change in the electron/hole quantum dot location as a function of a finely tuned twist angle can be considered as a crossover, and specifically for electrons, such a crossover involves an intermediate regime where the conduction band edge passes through a quantum wire-like domain wall. This result improves on a recently studied model^21^ based on a domain wall structure without twirling, which largely overestimated the amount of hydrostatic strain and, therefore, the depth of the quantum dot profiles. For all of these confinement regimes, we computed the binding energies for charge carriers (Figure 1 for AP-MoSe_2_/WSe_2_ and Supporting Information for AP-MoS_2_/WS_2_), taking into account the dielectric environment of hBN-encapsulated bilayers (which is relevant for the piezopotential contribution toward band edge profiles), and quantified the energies of terahertz-active intradot transitions (Figure 3). Additionally, the XX and MoW nodes feature peaks of a strain-induced pseudomagnetic field,^19,36^ which would split σ_+_ and σ_–_ polarized transitions for electrons/holes in K and K′ valleys, and we estimated them to be on the order of ∼1 meV.

In contrast, marginally twisted P bilayers feature quantum dots for both electrons and holes in the XX nodes of their DWN, though for holes such quantum dot confinement is quickly lost at twist angles θ[rad] > δ due to a carrier delocalization into triangular XW stacking domains (Figures 1 and 4), accompanied by reduction of the s–p on-dot orbital splitting (Figure 4).

**Figure 5:**
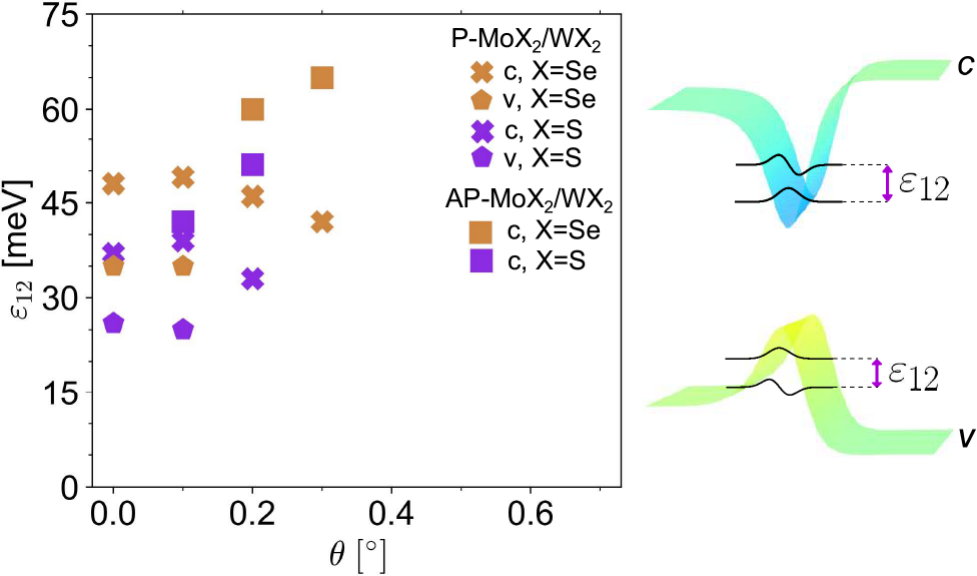
Subband splitting in 1D quantum wires associated with domain walls. Twist angle dependence of splitting, ε_12_, between the two lowest subbands for conduction (c) and valence (v) band electrons of AP- and P-MoX_2_/WX_2_ heterobilayers (orange or purple symbols for X = Se or S, respectively). Insets illustrate energy profiles for 1D energy channels and the corresponding subband wave functions.

We also note that boundaries between MoX and XW domains in P bilayers look like quantum wires, in particular for electrons. Such quantum wires can be characterized by two lowest subbands, ε_1_ and ε_2_. For AP bilayers, the 1D states at the domain wall appear to provide the band edge for electrons in the crossover regime discussed in Figure 1. For the sake of completeness, in Figure 5 we also show the intersubband splittings, ε_12_, for 1D domain wall states across a broader angle range for both P and AP structures (data for c and v subbands in P bilayers are marked by × and ⬟, respectively; subband splittings for the intermediate quantum wire regime for conduction band electrons in AP bilayers are marked by ■).

While this analysis was focused on single electron or hole states, it also suggests the effect of DWN on few particle complexes. For example, the simultaneous confinement of both electrons and holes at the XX nodes of triangular DWNs in P bilayers (promoted by hydrostatic strain) points toward interlayer exciton localization at those nodes in bilayers with θ[rad] < δ. This contrast to the charge (electron/hole) separation in the opposite DWN corners in AP bilayers across the same twist angle range. One may also speculate that the band edge profiles near the DWN in marginally twisted (both P and AP) bilayers would also confine X^–^ and X^+^ trions,^37−39^ with the same scenario as for electrons and holes, respectively.

## Supporting Information Available

Details about the lattice reconstruction analysis, DFT calculations, multiscale modeling of band edges, piezoelectric potential of encapsulated heterobilayers, calculation and structure of quantum dot states, and binding energies for quantum dots and wires in MoS_2_/WS_2_ (PDF)
