# Supplementary material for: Competition of Moiré Network Sites to Form Electronic Quantum Dots in Reconstructed MoX2/WX2 Heterostructures
Source: Nano Lett. 2024 Jan 31;24(6):1996–2002. doi: 10.1021/acs.nanolett.3c04427 (PMC10870774; doi:10.1021/acs.nanolett.3c04427)
Supplement: Supplementary file 1 — nl3c04427_si_001.pdf [file nl3c04427_si_001.pdf]

Supporting information:
Competition of moiré network sites to form electronic quantum dots in reconstructed MoX_2/WX_2 heterostructures

Isaac Soltero,[1,2,*] Mikhail A. Kaliteevski,[1,2] James G. McHugh,[1,2]
Vladimir Enaldiev,[1,2] and Vladimir I. Fal'ko[1,2,3,†]

[1] *Department of Physics and Astronomy, University of Manchester. Oxford Road, Manchester, M13 9PL, United Kingdom*
[2] *National Graphene Institute, University of Manchester. Booth St. E., Manchester, M13 9PL, United Kingdom*
[3] *Henry Royce Institute for Advanced Materials, University of Manchester. Oxford Road, Manchester, M13 9PL, United Kingdom*

Supplementary Note A. LATTICE RECONSTRUCTION ANALYSIS

As step (i) of the implemented multi-scale modelling (Methods section in the Main text), we computed 2D displacement fields $\mathbf{u}^{\mathrm{Mo/W}}(\mathbf{r})$ by minimizing the total energy of the bilayer over the moiré supercell, considering both elastic and adhesion energy contributions; this is done by solving a set of coupled Euler-Lagrange equations, in line with Refs. 1–3. Since the established domain wall widths are of the order of a few nanometers [1], a grid finesse of about 1 nm is required, leading to more than 10000 equations in the system. In order to achieve convergence of the numerical solution, we start from zero displacements, $\mathbf{u}^{\mathrm{Mo/W}} = 0$, for two decoupled monolayers, gradually scaling up interlayer adhesion energy from zero to its actual functional form determined using DFT computations in Ref. 2. By using the solution obtained at each previous iteration as a starting point for each next step, with an increased interlayer interaction we mimic the annealing of the rigidly incommensurate bilayer into a domain structure and determine the deformation fields across the DWN.

Supplementary Note B. DFT CALCULATIONS FOR BAND EDGES IN STRAINED MONOLAYERS

Several earlier DFT studies [4–7] of TMDs predicted a linear shift of conduction and valence band edges with increasing hydrostatic strain. This effect is stronger for K-point band edges as compared to Γ and Q-points. Therefore, we consider only the K-point band edges for conduction band in MoX_2 and valence band in WX_2, as the offset between band structures of MoX_2 and WX_2 suppresses the interlayer hybridization which otherwise would promote Γ and Q valleys to the band edge of a bilayer. Here, we also perform DFT calculations using the Quantum ESPRESSO code [8], with Vanderbilt ultrasoft pseudopotentials, under the generalized gradient approximation (GGA) to the exchange-correlation functional, as parameterised by Perdew, Burke and Ernzerhof. A plane-wave basis with a maximum cut-off of $E_w = 50$ Ry was used, with a charge density cut-off $E_\rho = 600$ Ry, and a Monkhorst-Pack k-point grid of dimensions $21 \times 21 \times 1$. Fermi-Dirac smearing of width $\sigma = 0.01$ eV was applied to aid convergence. Spin-orbit coupling was included in all band structure calculations. Calculations were performed to predict the variation in band edge position of strained TMD monolayers. Strain configurations were modelled through coordinate transformation of a monolayer unit cell,

$$U = \begin{pmatrix} 1 + u_{xx} & 0 \\ 0 & 1 + u_{yy} \end{pmatrix},$$

where $u_{ij} = (\partial_i u_j + \partial_j u_i)/2$ is a 2D strain tensor (so that $\mathrm{div}\,\mathbf{u}^{\mathrm{Mo/W}} \equiv u_{ii}^{\mathrm{Mo/W}}$), and $u_{xx} = u_{yy} \equiv u$. Values of the strain increment u in the range $\pm 2\%$ were used. For each value of u, lattice vectors were fixed in the deformed configuration, and atomic positions were structurally relaxed. Different orientations of the strain principal axis and TMD lattice vectors, as well as values of $u_{xx} \neq u_{yy}$, were also considered. In all cases, we find a linear shift of edges with respect to hydrostatic strain, leading to the parametrization shown in the Main text.

---

* isaac.solteroochoa@manchester.ac.uk
† vladimir.falko@manchester.ac.uk

Supplementary Note C. MULTI-SCALE MODELLING OF BAND EDGES

At step (ii) of the multi-scale modelling (Methods section in the Main text), we take into account that while, in each layer, hydrostatic strain shifts conduction and valence band edges in the same direction, the reversed sign of strain in MoX_2 and WX_2 layers leads to the opposite direction of band edge shifts at each point of the heterostructure. Inside the domains $\mathrm{div}\,\mathbf{u}^{\mathrm{Mo}}$ lifts up the K-point conduction band edge and $\mathrm{div}\,\mathbf{u}^{\mathrm{W}}$ pushes the valence band edge down, whereas a much larger strain with an opposite sign has the opposite effect on the band edges along the DWN, particularly XX nodes. We also take into account piezoelectric response of each of the involved TMDs to strain (step (iii)), leading to the on-layer piezoelectric charge densities,

$$\rho^{\mathrm{Mo/W}}(\mathbf{r}) = e_{11}^{\mathrm{Mo/W}}[2\partial_x u_{xy}^{\mathrm{Mo/W}}(\mathbf{r}) + \partial_y(u_{xx}^{\mathrm{Mo/W}}(\mathbf{r}) - u_{yy}^{\mathrm{Mo/W}}(\mathbf{r}))]. \tag{S1}$$

Here, $e_{11}^{\mathrm{Mo/W}}$ are piezocoefficients: $e_{11}^{\mathrm{Mo}} \approx e_{11}^{\mathrm{W}}$ for P and $e_{11}^{\mathrm{Mo}} \approx -e_{11}^{\mathrm{W}}$ for AP bilayers. As displacement fields and strain tensors in the two layers have the opposite signs in AP bilayers, the piezocharges add up and produce substantial piezoelectric potentials φ (calculated for an hBN encapsulated bilayer, see Supplementary Note D), in contrast to an almost exact compensation of piezocharges and negligible piezopotentials in P bilayers.

An important remark is that displacement fields are computed in a reference frame $x'\mathcal{O}y'$ which allows to fix periodic boundary conditions. This prime reference frame is rotated by an angle $\gamma = \pi/6 + \arctan[(\sqrt{3}\delta - \theta)/(\sqrt{3}\theta + \delta)]$ with respect to the fixed frame where x and y are along zigzag and armchair directions in the crystals, respectively, and in which Eq. (S1) is defined. Fig. S1 shows piezocharge distributions in the Mo layer for different twist angles in AP- and P-$MoSe_2$/WSe_2 bilayers. In AP bilayers, piezocharges show a concentration around domain walls and the node, maintaining charge neutrality in the 2H stacking domains. In this case, we get a finite contribution in the XX and MoW nodes, showing a change of sign around $\theta \approx \delta$. In P bilayers, the piezocharge distribution shows opposite sign for MoX and XW stacking domains, with a tendency towards concentration around the charge neutral domain walls and nodes. Additionally, there is a sign switch in the charge density in these domains when crossing $\theta \approx \delta$.

Finally, the interlayer hybridisation gives rise to a weak interlayer polarization (double charge layer at the interface), which produces an additional stacking-dependent interlayer energy shifts $\Delta(\mathbf{r})$. This energy contribution is described by the interpolation formula

$$\Delta^{\mathrm{AP/P}}(\mathbf{r}) = \Delta_0[d(\mathbf{r})] + \Delta_a^{\mathrm{AP/P}}[d(\mathbf{r})] \sum_{n=1}^{3} \sin\left(\mathbf{G}_n \cdot \mathbf{r}_0\right). \tag{S2}$$

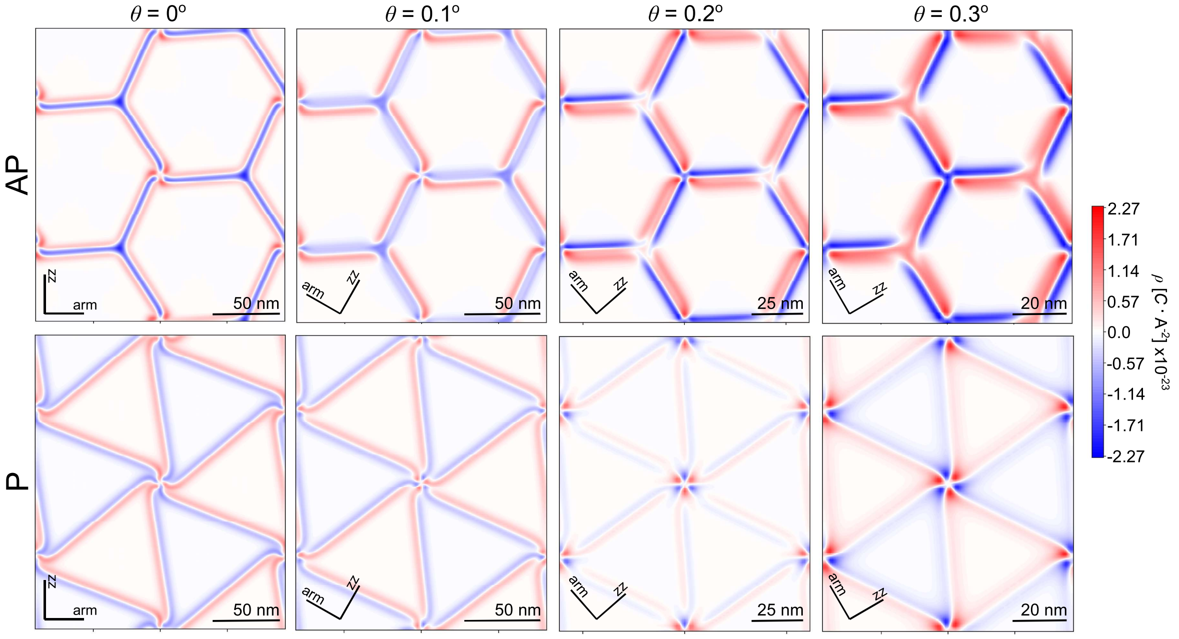

FIG. S1. Piezoelectric charge density maps in the Mo layer in AP- (top) and P-$MoSe_2$/WSe_2 (bottom) for multiple twist angles.

where $\mathbf{G}_{1,2,3}$ are three vectors related to the first Bragg star of the monolayer crystals. Full expressions for Δ_0, Δ_a^{P} ($\Delta_a^{\mathrm{AP}} = 0$) and the interlayer distance modulation $d(\mathbf{r})$ can be found in Ref. 2. We assume that the energy contribution is equally distributed between both layers, leading to a $\pm\Delta/2$ term in the band edge equation.

Altogether, the effect of hydrostatic strain and the above described electrostatics are incorporated in the band energy profiles,

$$\delta\varepsilon_{c/v}(\mathbf{r}) = \mathcal{V}_{c/v}\,\mathrm{div}\,\mathbf{u}^{\mathrm{Mo/W}}(\mathbf{r}) - e\varphi_{c/v}(\mathbf{r}) \mp \frac{1}{2}\Delta(\mathbf{r}). \tag{S3}$$

as shown in the Main text. In Figs. S2 and S3 we show the spatial distribution of the relevant contributions in Eq. (S3) to the conduction band edge shifts for multiple twist angles in AP-$MoSe_2/WSe_2$ and P-$MoSe_2/WSe_2$, respectively.

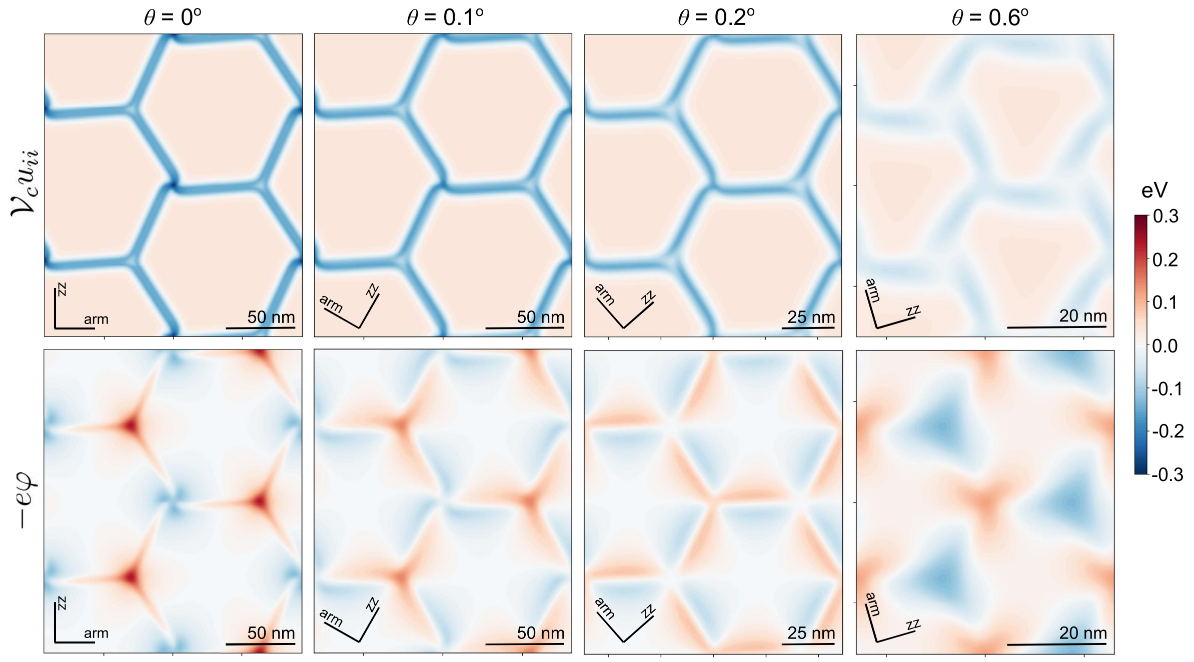

FIG. S2. Hydrostatic strain (top) and piezopotential (bottom) energy contributions for the conduction band spatial modulation in AP-$MoSe_2$/WSe_2 for multiple twist angles.

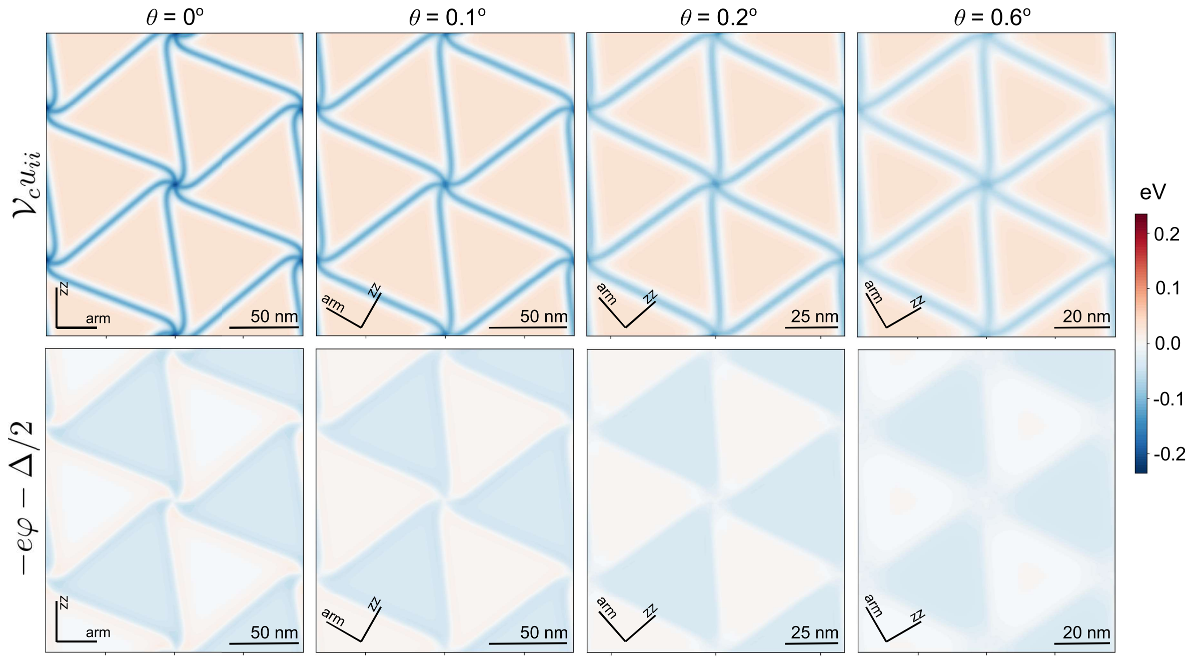

FIG. S3. Hydrostatic strain (top) and joint piezopotential and interlayer charge transfer (bottom) energy contributions for the conduction band spatial modulation in P-$MoSe_2$/WSe_2 for multiple twist angles.

Supplementary Note D. PIEZOELECTRIC POTENTIAL FOR ENCAPSULATED HETEROBILAYERS

The electric potential $\varphi(\mathbf{r}, z)$ produced by the piezoelectric charges induced in both layers is calculated via the Green's function method for the Poisson equation. We will consider an heterobilayer with hBN encapsulation (see Fig. S4), where we label the Mo layer as the top layer (t) and the W layer as the bottom layer (b). The piezopotential φ due to piezocharge density in both layers satisfies the equation

$$\left[\varepsilon_{\parallel}(z)\nabla_{\mathbf{r}}^2 + \varepsilon_{\perp}(z)\partial_z^2 + 4\pi \sum_{l'=t,b} \alpha_{2\mathrm{D}}^{l'} \delta(z - z_{l'})\nabla_{\mathbf{r}}^2\right]\varphi(\mathbf{r}, z) = -4\pi\left[\rho_t(\mathbf{r})\delta(z - z_t) + \rho_b(\mathbf{r})\delta(z - z_b)\right] \tag{S4}$$

where

$$\varepsilon_{\parallel/\perp}(z) = \begin{cases} \varepsilon_{\parallel/\perp}, & \text{if } z < z_2 \text{ or } z > z_1 \\ 1, & \text{if } z_2 < z < z_1 \end{cases}. \tag{S5}$$

In Eq. (S5), $\varepsilon_{\parallel} = 6.9$ and $\varepsilon_{\perp} = 3.7$ are the in- and out-of-plane dielectric permitivities of bulk hBN crystals [9, 10], and $\alpha_{2\mathrm{D}}^{t/b}$ is the in-plane electric polarizability for each monolayer. Due to the in-plane translational invariance, the Green's function related to Eq. (S4) is given by

$$\left[\varepsilon_{\parallel}(z)\nabla_{\mathbf{r}}^2 + \varepsilon_{\perp}(z)\partial_z^2 + 4\pi \sum_{l'=t,b} \alpha_{2\mathrm{D}}^{l'} \delta(z - z_{l'})\nabla_{\mathbf{r}}^2\right] G(\mathbf{r} - \mathbf{r}'; z, z') = \delta(\mathbf{r} - \mathbf{r}')\delta(z - z'). \tag{S6}$$

Taking the Fourier transform over $\mathbf{r} - \mathbf{r}'$ gives

$$\varepsilon_{\perp}(z)\partial_z^2 G(\mathbf{q}; z, z') - \varepsilon_{\parallel}(z) q^2 G(\mathbf{q}; z, z') - 4\pi q^2 \sum_{l'=t,b} \alpha_{2\mathrm{D}}^{l'} \delta(z - z_{l'}) G(\mathbf{q}; z, z') = \delta(z - z'). \tag{S7}$$

Solving Eq. (S7) in each region and applying the appropriate boundary conditions at the interfaces, we obtain the Green's function in z due to a source in z'. We are interested in the cases where $z = z_{t/b}$, considering the contributions of source terms in each layer. The obtained Green's functions are

$$G(\mathbf{q}; z_t, z_{t/b}) = \frac{F_t^{(1)}(q) e^{\mp\frac{1}{2} q d_0} + F_t^{(2)}(q) e^{\pm\frac{1}{2} q d_0}}{I(q)}, \tag{S8a}$$

$$G(\mathbf{q}; z_b, z_{t/b}) = \frac{F_b^{(1)}(q) e^{\mp\frac{1}{2} q d_0} + F_b^{(2)}(q) e^{\pm\frac{1}{2} q d_0}}{I(q)}, \tag{S8b}$$

with

$$F_t^{(1)}(q) = e^{\frac{q d_0}{2}}\left[1 - \varepsilon' + (1 + \varepsilon') e^{2qd'}\right]\left[-1 + \varepsilon' - 2\pi q \alpha_{2\mathrm{D}}^b(-1 + \varepsilon') + 2\pi q \alpha_{2\mathrm{D}}^b (1 + \varepsilon') e^{2d'q}\right], \tag{S9a}$$

$$F_t^{(2)}(q) = e^{\frac{3}{2} d_0 q}\left[-2\pi q \alpha_{2\mathrm{D}}^b(-1 + \varepsilon')^2 - (1 + \varepsilon')^2 (1 + 2\pi q \alpha_{2\mathrm{D}}^b) e^{4d'q} + (-1 + \varepsilon'^2)(1 + 4\pi q \alpha_{2\mathrm{D}}^b) e^{2d'q}\right], \tag{S9b}$$

$$F_b^{(1)}(q) = e^{\frac{3}{2} d_0 q}\left[-2\pi q \alpha_{2\mathrm{D}}^t(-1 + \varepsilon')^2 - (1 + \varepsilon')^2 (1 + 2\pi q \alpha_{2\mathrm{D}}^t) e^{4d'q} + (-1 + \varepsilon'^2(1 + 4\pi q \alpha_{2\mathrm{D}}^t) e^{2d'q})\right], \tag{S9c}$$

$$F_b^{(2)}(q) = e^{\frac{1}{2} d_0 q}\left[1 - \varepsilon' + (1 + \varepsilon') e^{2d'q}\right]\left[-1 + \varepsilon' - 2\pi q \alpha_{2\mathrm{D}}^t(-1 + \varepsilon') + 2\pi q \alpha_{2\mathrm{D}}^t (1 + \varepsilon') e^{2d'q}\right], \tag{S9d}$$

$$\begin{aligned} I(q) = \; & 2q\Big[-1 + 2\pi q \alpha_{2\mathrm{D}}^b + (1 + 2\alpha_{2\mathrm{D}}^b \pi q)(1 + 2\pi q \alpha_{2\mathrm{D}}^t) e^{2(d_0 + 2d')q} + 2\varepsilon'\Big\{1 - 2\pi q \alpha_{2\mathrm{D}}^b \\ & - 2\pi q \alpha_{2\mathrm{D}}^t\left[1 + 2\pi q \alpha_{2\mathrm{D}}^b(-1 + e^{2d_0 q} + e^{4d'q})\right] + (1 + 2\pi q \alpha_{2\mathrm{D}}^b)(1 + 2\pi q \alpha_{2\mathrm{D}}^t) e^{2(d_0 + 2d')q}\Big\} \\ & + 2\pi q\Big\{\alpha_{2\mathrm{D}}^b e^{2d'q} + \alpha_{2\mathrm{D}}^t + \alpha_{2\mathrm{D}}^t e^{2d'q} - 2\pi q \alpha_{2\mathrm{D}}^b \alpha_{2\mathrm{D}}^t + 2\pi q \alpha_{2\mathrm{D}}^b \alpha_{2\mathrm{D}}^t e^{2d_0 q} - 4\pi q \alpha_{2\mathrm{D}}^b \alpha_{2\mathrm{D}}^t e^{2d'q} \\ & - 2\pi q \alpha_{2\mathrm{D}}^b \alpha_{2\mathrm{D}}^t e^{4d'q} + (\alpha_{2\mathrm{D}}^b + \alpha_{2\mathrm{D}}^t + 4\pi q \alpha_{2\mathrm{D}}^b \alpha_{2\mathrm{D}}^t) e^{2(d_0 2d')q}\Big\} + \varepsilon'^2\Big\{-1 + 2\pi q \alpha_{2\mathrm{D}}^b \\ & + (1 + 2\pi q \alpha_{2\mathrm{D}}^b)(1 + 2\pi q \alpha_{2\mathrm{D}}^t) e^{2(d_0 + 2d')q} + 2\pi q\big[\alpha_{2\mathrm{D}}^t - \alpha_{2\mathrm{D}}^t(1 + e^{2d_0 q}) e^{2d'q} + \alpha_{2\mathrm{D}}^b(-2\pi q \alpha_{2\mathrm{D}}^t \\ & + 2\pi q \alpha_{2\mathrm{D}}^t e^{2d_0 q} - 2\pi q \alpha_{2\mathrm{D}}^t e^{4d'q} + (-1 + 4\pi q \alpha_{2\mathrm{D}}^t) e^{2d'q} - (1 + 4\pi q \alpha_{2\mathrm{D}}^t) e^{2(d_0 + 2d')q})\big]\Big\}\Big], \end{aligned} \tag{S9e}$$

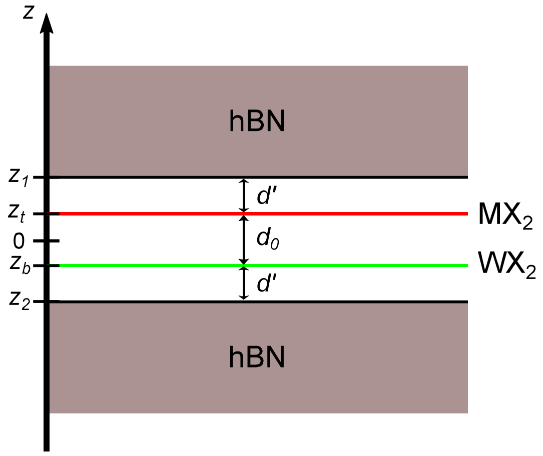

FIG. S4. System scheme for heterobilayer encapsulated in hBN.

where we have defined $\varepsilon' = \sqrt{\varepsilon_{\parallel}/\varepsilon_{\perp}}$. The piezoelectric potential is given by the convolution

$$\varphi(\mathbf{r}, z_l) = -4\pi \int d^2r' \Big[G(\mathbf{r}-\mathbf{r}'; z_l, z_t)\rho_t(\mathbf{r}') + G(\mathbf{r}-\mathbf{r}'; z_l, z_b)\rho_b(\mathbf{r}')\Big], \tag{S10}$$

or equivalently, it can be expressed as the inverse Fourier transform of the function

$$\tilde{\varphi}(\mathbf{q}, z_l) \equiv -4\pi\Big[G(\mathbf{q}; z_l, z_t)\tilde{\rho}_t(\mathbf{q}) + G(\mathbf{q}; z_l, z_b)\tilde{\rho}_b(\mathbf{q})\Big], \tag{S11}$$

where

$$\tilde{\rho}(\mathbf{q}) \equiv \int d^2r\, e^{-i\mathbf{q}\cdot\mathbf{r}}\, \rho(\mathbf{r}). \tag{S12}$$

The piezopotential in real space is obtained through the Fourier series expansion of the charge density in terms of the moiré reciprocal lattice vectors $\mathbf{g}_n$,

$$\rho_l(\mathbf{r}) = \sum_{n=1}^{\infty} \Big[\rho^l_{n,s}\cos(\mathbf{g}_n\cdot\mathbf{r}) + \rho^l_{n,a}\sin(\mathbf{g}_n\cdot\mathbf{r})\Big], \tag{S13}$$

such that its corresponding Fourier transform is

$$\tilde{\rho}_l(\mathbf{q}) = \frac{(2\pi)^2}{2}\sum_{n=1}^{\infty}\Big[(\rho^l_{n,s} - i\rho^l_{n,a})\delta(\mathbf{q}-\mathbf{g}_n) + (\rho^l_{n,s} + i\rho^l_{n,a})\delta(\mathbf{q}+\mathbf{g}_n)\Big]. \tag{S14}$$

Substituting (S14) in Eq. (S11) and applying an inverse Fourier transform, we obtain the piezopotential in layer l,

$$\begin{aligned}\varphi(\mathbf{r}, z_l) = -4\pi\sum_{n=1}^{\infty}\Big[&\big\{G(\mathbf{g}_n; z_l, z_t)\rho^t_{n,s} + G(\mathbf{g}_n; z_l, z_b)\rho^b_{n,s}\big\}\cos(\mathbf{g}_n\cdot\mathbf{r})\\ &+ \big\{G(\mathbf{g}_n; z_l, z_t)\rho^t_{n,a} + G(\mathbf{g}_n; z_l, z_b)\rho^b_{n,a}\big\}\sin(\mathbf{g}_n\cdot\mathbf{r})\Big].\end{aligned} \tag{S15}$$

Supplementary Note E. CALCULATION OF QUANTUM DOT BOUND STATES

As step (iv) of the multi-scale (Methods in the Main text), we analyse the vicinity of DWN nodes that have a quantum dot profile by representing the energy profiles in the form of angular harmonics,

$$\delta\varepsilon^{\rm QD}_{c/v}(\mathbf{r}) = A_0^{c/v}(r) + \sum_{\ell=1}^{N}\left[A_\ell^{c/v}(r)\cos(\ell\phi) + B_\ell^{c/v}(r)\sin(\ell\phi)\right]. \tag{S16}$$

Here, we use polar coordinates centered at the node, r and ϕ, $A_\ell^{\rm Mo/W}(r)$ and $B_\ell^{\rm Mo/W}(r)$ are coefficients that depend only on the distance to the center of the network node (at $\mathbf{r} = \mathbf{0}$). As a check for the accuracy of determining coefficients $A_\ell^{\rm Mo/W}$ and $B_\ell^{\rm Mo/W}$ numerically from the computed band edge landscapes, we use the fact that, for both AP and P bilayers, the C_3 symmetry is maintained around the nodes, so that the expansion of $\delta\varepsilon^{\rm QD}_{c/v}$ would only contain harmonics with $\ell = 3n$. In this computation, a cut-off N was established upon convergence to the computed band edges profiles, for which good agreement was found by truncating up to $N = 36$.

Then, we find spectral characteristics of the quantum dot states for conduction and valence bands by numerical diagonalization of the Hamiltonian,

$$H_{c/v} = \frac{\mathbf{p}^2}{2m_{c/v}} + \delta\varepsilon^{\rm QD}_{c/v}(\mathbf{r}), \tag{S17}$$

where effective masses are [11]: $m_c = 0.49m_0$, $m_v = -0.36m_0$ for $MoSe_2/WSe_2$, and $m_c = 0.44m_0$, $m_v = -0.35m_0$ for MoS_2/WS_2 (m_0 is the free electron mass). We adopt a direct diagonalization approach using the following orthogonal basis [12],

$$\langle\mathbf{r}|n,m\rangle \equiv f_{nm}\left(\frac{r}{\lambda}\right)\frac{e^{im\phi}}{\sqrt{2\pi}},$$

$$f_{nm}(\xi) = \sqrt{\frac{2\,\Gamma(\frac{n-|m|}{2}+1)}{\lambda^2\,\Gamma(\frac{n+|m|}{2}+1)}}\,\xi^{|m|}e^{-\xi^2/2}L^{|m|}_{\frac{n-|m|}{2}}(\xi^2),$$

where $m \in \mathbb{Z}$ is the angular momentum quantum number, $L_n^m(x)$ are associated Laguerre polynomials. For each quantum number $n \in \mathbb{N}_0$, the angular momentum is restricted by $|m| \le n$ and $(n - |m|) \bmod 2 = 0$. Matrix elements for the first term in (S17) can be computed by introducing the 2D harmonic oscillator Hamiltonian H_0, for which the basis elements are eigenstates. This gives

$$\begin{aligned}\langle n',m'|\frac{\mathbf{p}^2}{2m_{c/v}}|n,m\rangle &= \langle n',m'|H_0|n,m\rangle - \frac{1}{2m_{c/v}}\left(\frac{\hbar}{\lambda^2}\right)^2\langle n',m'|r^2|n,m\rangle \\ &= \delta_{n,n'}\delta_{m,m'}\frac{\hbar^2(n+1)}{m_{c/v}\lambda^2} - \frac{\delta_{m,m'}}{2m_{c/v}}\left(\frac{\hbar}{\lambda^2}\right)^2\langle n',m|r^2|n,m\rangle,\end{aligned} \tag{S18}$$

where [13]

$$\begin{aligned}\langle n',m|r^2|n,m\rangle &= \sqrt{\frac{4(\frac{n'-|m|}{2})!(\frac{n-|m|}{2})!}{\lambda^4(\frac{n'+|m|}{2})!(\frac{n+|m|}{2})!}}\int_0^\infty dr\, r^3\left(\frac{r}{\lambda}\right)^{2|m|}e^{-\frac{r^2}{\lambda^2}}L^{|m|}_{\frac{n'-|m|}{2}}\left(\frac{r^2}{\lambda^2}\right)L^{|m|}_{\frac{n-|m|}{2}}\left(\frac{r^2}{\lambda^2}\right) \\ &= \frac{\lambda^2(|m|+1)}{(|m|)!}\sqrt{\frac{(\frac{n'+|m|}{2})!(\frac{n+|m|}{2})!}{(\frac{n'-|m|}{2})!(\frac{n-|m|}{2})!}}\sum_{j=0}^{\frac{n'-|m|}{2}}\frac{(\frac{|m|-n'}{2})_j(|m|+2)_j}{(|m|+1)_j\,j!}\sum_{k=0}^{\frac{n-|m|}{2}}\frac{(\frac{|m|-n}{2})_k(j+|m|+2)_k}{(|m|+1)_k\,k!}.\end{aligned} \tag{S19}$$

Matrix elements related to the quantum dot profiles are

$$\begin{aligned}\langle n',m'|\delta\varepsilon^{\rm QD}|n,m\rangle = \frac{1}{2}\sum_{\ell=0}^{N}\Bigg[&\big(\delta_{m,m'+\ell}+\delta_{m,m'-\ell}\big)\int_0^\infty dr\, r f^*_{n'm'}\left(\frac{r}{\lambda}\right)f_{nm}\left(\frac{r}{\lambda}\right)A_\ell(r) \\ &+ i\big(\delta_{m,m'+\ell}-\delta_{m,m'-\ell}\big)\int_0^\infty dr\, r f^*_{n'm'}\left(\frac{r}{\lambda}\right)f_{nm}\left(\frac{r}{\lambda}\right)B_\ell(r)\Bigg],\end{aligned} \tag{S20}$$

where integrals are computed numerically. Due to the C_3 symmetry of the quantum dot profiles in (S3), the matrix form of the Hamiltonian is block diagonal, coupling states with $m' - m = \pm 3\ell$. This allows to identify two types of lowest energy states: one s-like states, based on $m = 3\ell$ harmonics, and the other a doublet of p-like states based on $m = 3\ell \pm 1$ orbitals.

The numerical diagonalization of the eigenvalue problem can be performed by fixing the parameter λ and maximum value for the principal quantum number $n_{\max}$ in the basis functions. To optimize this values, we first solve the eigenvalue problem for multiple values of λ, ranging from 10 to 40 Å with $n_{\max} = 20$. In Fig. S5(a) we show the ground state for $\bar{m} = 0$ and $\bar{m} = \pm 1$ in P-$MoSe_2$/WSe_2 with $\theta = 0°$ as a function of λ, from which we find that $\lambda_* = 20$ Å minimizes the ground state energy. After this, we solve the eigenvalue problem increasing the value $n_{\max}$ until we find convergence of the lowest energy level for $\bar{m} = 0, \pm 1$ within a 0.5 meV tolerance (Fig. S5(b)). For $\bar{m} = \pm 1$, convergence is found at larger values of $n_{\max}$ compared to $\bar{m} = 0$ since this have a larger spatial extension and a stronger mixing with higher angular momentum basis elements.

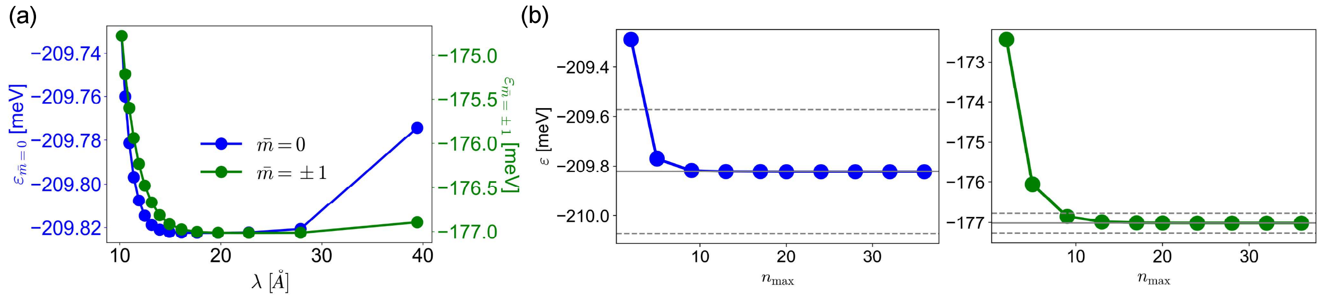

FIG. S5. (a) Lowest energy level for quantum dots in P-$MoSe_2$/WSe_2 with $\theta = 0°$ as a function of λ and a maximum value $n_{\max} = 20$. Results are shown for effective angular momentum $\bar{m} = 0$ (blue) and $\bar{m} \pm 1$ (green). (b) Convergence of the lowest energy level with respect to $n_{\max}$ and a fixed value of $\lambda = 20$ Å. Solid lines show the converged energy and dashed lines define a ± 0.25 meV range centered at this value.

Supplementary Note F. QUANTUM DOTS AND WIRES IN MoS_2/WS_2

In this section we show energies for all bound states in quantum dots and wires for AP- and P-MoS_2/WS_2 with $0° \leq \theta \leq 0.7°$.

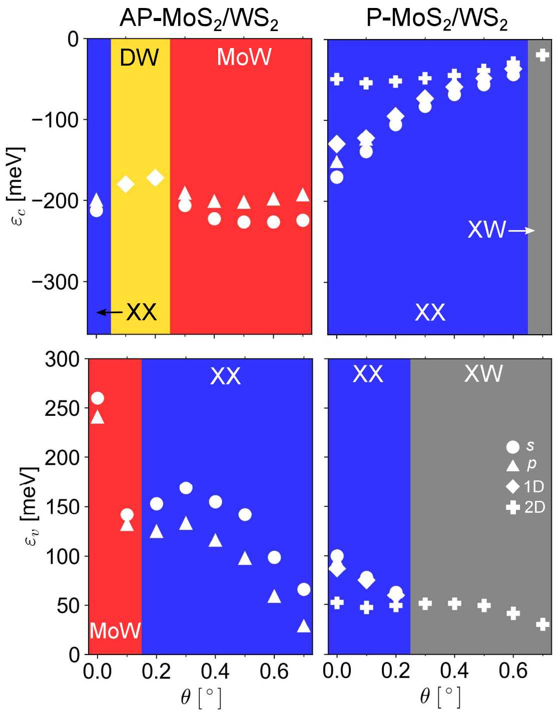

FIG. S6. Localization diagram for electrons and holes in AP- (left) and P-MoS_2/WS_2 (right) bilayers with small twist angle. Color code illustrates the location of the lowest energy for conduction band electrons (top) and valence band holes (bottom) as a function of twist angle. White symbols indicate energies for quantum dots (s and p), wires along domain walls (1D), and XW domain boxes (2D).

Supplementary Note G. STRUCTURE OF QUANTUM DOT BOUND STATES

In this section we analyze the spatial extension of the lowest energy quantum dot states. To quantify the radial spreading of the functions, we compute the standard deviation $\sigma_r(1s) \equiv \sqrt{\langle r^2 \rangle_s - \langle r \rangle_s^2}$ for s states in the XX and MoW nodes for the conduction and valence band edges (Fig. S7). We note that, for AP bilayers, wavefunctions show an increase in size for twist angles near the transition in quantum dot localization (from XX to MoW nodes in the conduction band and from MoW to XX nodes in the valence band). For both conduction and valence band states, the spatial extension of the states ranges between 10 and 20 Å. In P bilayers, the spatial extension of the wavefunctions increases linearly with the twist angle between layers, where the weaker confinement for the valence states is clearly reflected as a larger spatial extension compared to the conduction states. Additionally, as representative cases, we show the squared moduli of s and p states for the conduction and valence band states in aligned AP- and P-$MoSe_2/WSe_2$ in Fig. S8.

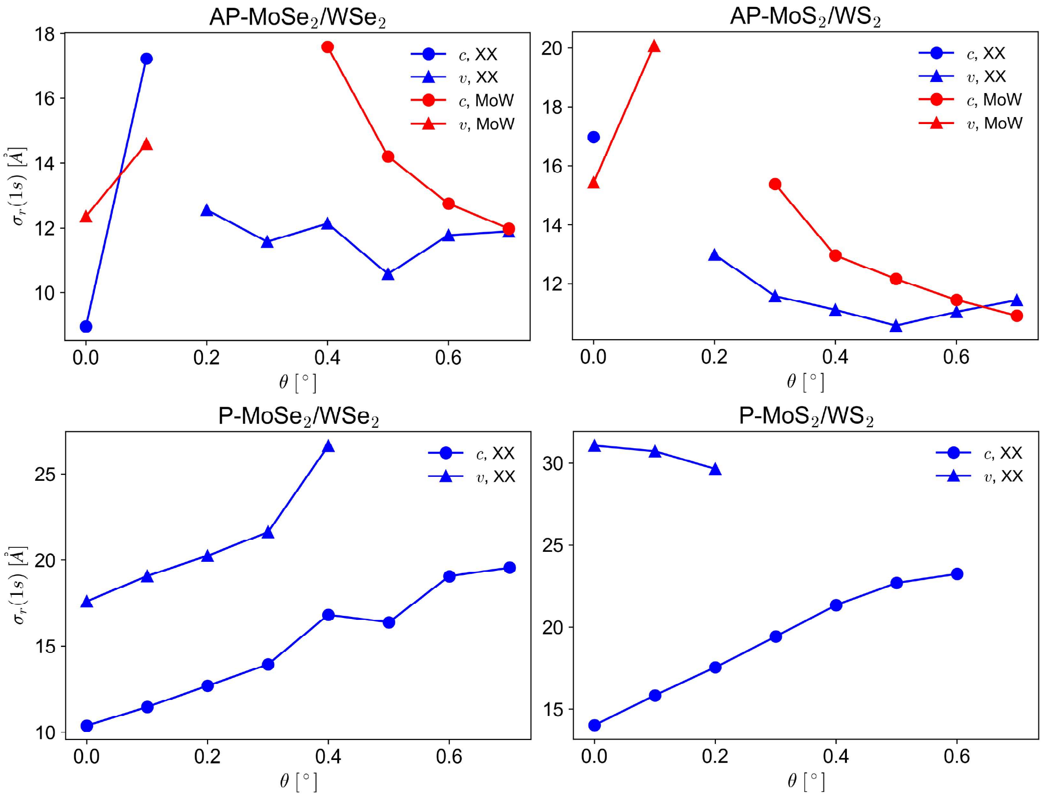

FIG. S7. Twist angle dependence of the radial standard deviation, σ_r, for quantum dot s states in the conduction (c) and valence (v) band edges in AP- (marked as circles) and P-MoX_2/WX_2 (marked as triangles). Different colours are used for quantum dots in the XX and MoW nodes.

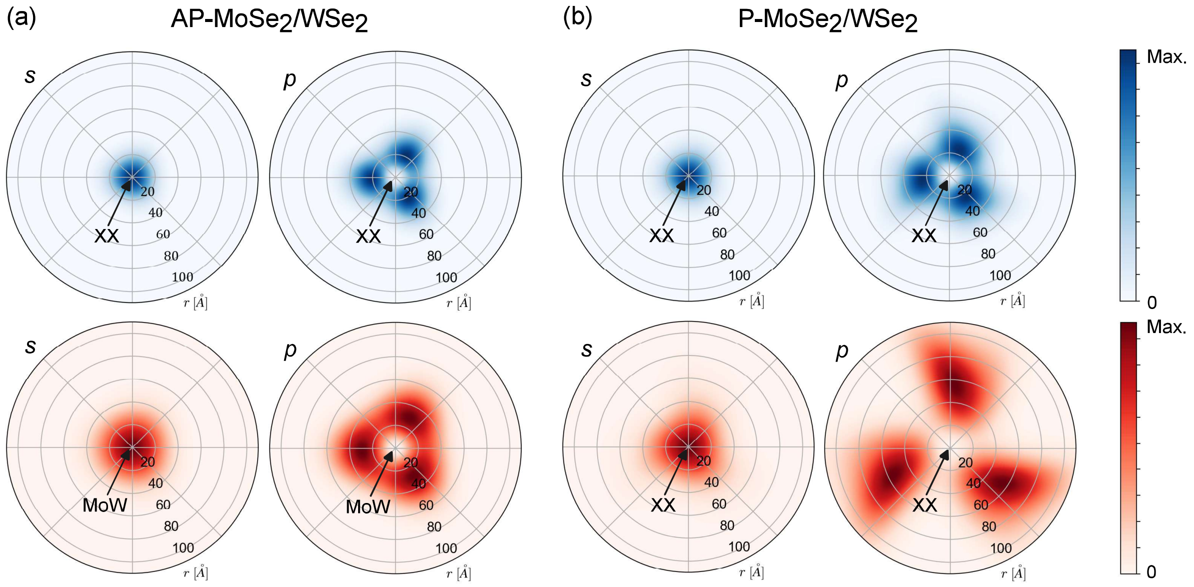

FIG. S8. Squared moduli of s and p states for the conduction (blue maps) and valence (red maps) band quantum dots in (a) AP- and (b) P-$MoSe_2/WSe_2$ with $\theta = 0°$. Each function is plotted in a 100 Å radius area around the corresponding localization node.

[1] V. Enaldiev, V. Zolyomi, C. Yelgel, S. Magorrian, and V. Fal'ko, Stacking domains and dislocation networks in marginally twisted bilayers of transition metal dichalcogenides, Physical Review Letters **124**, 206101 (2020).

[2] V. Enaldiev, F. Ferreira, S. Magorrian, and V. I. Fal'ko, Piezoelectric networks and ferroelectric domains in twistronic superlattices in WS_2/MoS_2 and $WSe_2/MoSe_2$ bilayers, 2D Materials **8**, 025030 (2021).

[3] M. A. Kaliteevski, V. Enaldiev, and V. I. Fal'ko, Twirling and spontaneous symmetry breaking of domain wall networks in lattice-reconstructed heterostructures of two-dimensional materials, Nano Letters **23**, 8875 (2023).

[4] H. J. Conley, B. Wang, J. I. Ziegler, R. F. Haglund Jr, S. T. Pantelides, and K. I. Bolotin, Bandgap engineering of strained monolayer and bilayer MoS_2, Nano letters **13**, 3626 (2013).

[5] K. P. Dhakal, S. Roy, H. Jang, X. Chen, W. S. Yun, H. Kim, J. Lee, J. Kim, and J.-H. Ahn, Local strain induced band gap modulation and photoluminescence enhancement of multilayer transition metal dichalcogenides, Chemistry of Materials **29**, 5124 (2017).

[6] K. Zollner, P. E. F. Junior, and J. Fabian, Strain-tunable orbital, spin-orbit, and optical properties of monolayer transition-metal dichalcogenides, Physical Review B **100**, 195126 (2019).

[7] V. Enaldiev, F. Ferreira, J. McHugh, and V. I. Fal'ko, Self-organized quantum dots in marginally twisted $MoSe_2/WSe_2$ and MoS_2/WS_2 bilayers, npj 2D Materials and Applications **6**, 74 (2022).

[8] P. Giannozzi, S. Baroni, N. Bonini, M. Calandra, R. Car, C. Cavazzoni, D. Ceresoli, G. L. Chiarotti, M. Cococcioni, I. Dabo, *et al.*, QUANTUM ESPRESSO: a modular and open-source software project for quantum simulations of materials, Journal of physics: Condensed matter **21**, 395502 (2009).

[9] R. Geick, C. Perry, and G. Rupprecht, Normal modes in hexagonal boron nitride, Physical Review **146**, 543 (1966).

[10] A. Laturia, M. L. Van de Put, and W. G. Vandenberghe, Dielectric properties of hexagonal boron nitride and transition metal dichalcogenides: from monolayer to bulk, npj 2D Materials and Applications **2**, 1 (2018).

[11] A. Kormányos, G. Burkard, M. Gmitra, J. Fabian, V. Zólyomi, N. D. Drummond, and V. Fal'ko, k·p theory for two-dimensional transition metal dichalcogenide semiconductors, 2D Materials **2**, 022001 (2015).

[12] D. A. Ruiz-Tijerina, I. Soltero, and F. Mireles, Theory of moiré localized excitons in transition metal dichalcogenide heterobilayers, Physical Review B **102**, 195403 (2020).

[13] G. Andreus, R. Askey, and R. Roy, *Special Functions, Encyclopedia of Mathematics and Its Applications, Vol. 71* (Cambridge University Press, Cambridge, 1999).
